# Two-layer synchronized ternary quantum-dot cellular automata wire crossings

**DOI:** 10.1186/1556-276X-7-221

**Published:** 2012-04-16

**Authors:** Iztok Lebar Bajec, Primož Pečar

**Affiliations:** 1 Faculty of Computer and Information Science, University of Ljubljana, Tržaška cesta, Slovenia

**Keywords:** Quantum-dot cellular automata, Ternary processing, Wire crossing, Multi-layer design, 85.35.Be, MCS: 68Q80, 03B50

## Abstract

Quantum-dot cellular automata are an interesting nanoscale computing paradigm. The introduction of the ternary quantum-dot cell enabled ternary computing, and with the recent development of a ternary functionally complete set of elementary logic primitives and the ternary memorizing cell design of complex processing structures is becoming feasible. The specific nature of the ternary quantum-dot cell makes wire crossings one of the most problematic areas of ternary quantum-dot cellular automata circuit design. We hereby present a two-layer wire crossing that uses a specific clocking scheme, which ensures the crossed wires have the same effective delay.

## Background

Since the first introduction of quantum-dot cellular automata (QCA), an interesting nanoscale computing paradigm by Lent et al. in 1993 [1], many researchers have embraced its simple concept and potential as a future processing platform [2-6]. In recent years, a group of researchers have presented a generalization of the basic QCA cell, namely the ternary QCA (tQCA) cell [7-10], which enables ternary computation. Their principal motivator was the premise that future processing platforms should not disregard the advantages of multi-valued processing [11-15]. The group presented the basic ternary building blocks, the inverter, majority gate, wire, corner wire and fan-out, and more recently also, a functionally complete set of ternary logic functions, based on Post Logic, and a memorizing tQCA circuit [16,17]. Due to the specifics of the tQCA cell, wire crossings seem to be the principal drawback before a more widespread acceptance of tQCA circuitry. Wire crossings are one of the most used steps in systematic logic design. In the classic, binary QCAs, wires can be crossed either in a coplanar fashion by using rotated QCA cells for one of the wires or in a multilayer fashion where two intermediate layers are used to prevent any possible crosstalk between the two crossing lines [18]. Although the multilayer approach proves to be more robust [19], the majority of designs employ the coplanar one; that is in fact one of the most praised features of classic QCA. Since coplanar crossings tend to be prone to robustness issues, much research has been devoted to its increase, even to the extents of altering the design of QCA logic gates [20-22], or as in the case of molecular implementations, through the elimination of crossings by logic gate duplication [23,24]. Another approach exploits the pipelined nature of QCAs and uses parallel-to-serial converters and a specialized clocking scheme to design a coplanar crossbar network [25]. Coplanar crossings with rotated tQCA cells are not possible, but multilayer crossings are, as it has been reported recently [26]. Here, we go a step further by presenting a wire crossing that is synchronized, i.e., the two wires employ such clocking schemes that the outputs of the two wires have the same effective delay. In addition, the clocking schemes allow for a two-layer design, in other words, removing the requirement for additional layers, whose sole purpose is to prevent possible crosstalk. The article is organized as follows. We first present the overview of a ternary QCA cell, its architecture and inter-cell interaction. We follow by presenting a two-layer design, where we first show the inter-layer interaction followed by presenting the design and clocking scheme of a two-layer synchronized wire crossing.

### The ternary QCA cell

**Figure 1:**
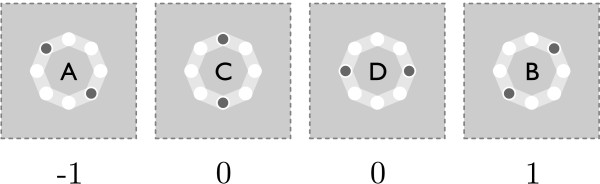
**Ternary quantum-dot cell (tQCA cell) ground states.** In the case of an isolated tQCA cell, a circular arrangement of quantum dots with two mobile electrons, there are four electron arrangements that ensure their maximal separation thus achieving a ground state. They are marked **A**, **B**, **C** and **D** and can be mapped to balanced ternary values -1, 0, and 1.

A QCA is, in general, a planar array of quantum-dot cells (QCA cells) [1]. In the case of a ternary QCA, it is an array of tQCA cells. A tQCA cell [7,8] is a circular arrangement of eight quantum-dots and two mobile electrons. The electrons tend to localize only in the quantum dots or tunnel between adjacent quantum dots, and they cannot tunnel outside of the cell. The Coulomb interaction causes the electrons to localize in the quantum dots that ensure their maximal separation (thus achieving the minimal energetic state). The four arrangements, which in the case of an isolated cell, correspond to the energetic minimal state (ground state) are marked as A, B, C, and D (see Figure 1).

It turns out that placing a well-polarized cell (i.e., a cell with electrons fixed in one of the four arrangements) nearby causes one of the four states to become the favored one even in the observed cell. When the well-polarized cell is on the same plane, but to the left, right, above, or below the observed cell, then the state A or B in the well-polarized cell induces the same state even in the observed cell. However, state C induces state D, and the same goes for state D, which induces state C in the observed cell. If one interprets state A, B, C, and D as balanced ternary logic values, so that state A represents logic value −1, state B logic value 1, and states C and D both logic value 0, then inter-cell interaction causes the logic value of the well-polarized cell to be transferred to the observed cell.

If the well-polarized cell is placed diagonally to the observed cell, then state A in the well-polarized cell induces state B in the observed cell, state B induces state A, state C induces state C, and state D induces state D. When the states are interpreted as logic values, this translates to logic negation. With specific planar arrangements of cells, it is thus possible to mimic the behavior of interconnecting wires as well as logic gates [27]. By interconnecting such building blocks, more complex devices capable of processing can be constructed.

The reliability of the logic value transfer throughout a QCA device (i.e., a spatial arrangement of QCA cells) depends foremost on the reliability of the switching process, i.e., the transition of a cell’s state that corresponds to one logic value to a state that corresponds to another. The reliability is ensured via the adiabatic switching concept [9,28], where a cyclic signal, namely adiabatic clock, is used to control the switching dynamics. The cyclic signal is comprised of four phases. The switch phase serves the cell’s gradual update of the state with respect to the neighbors. The hold phase is intended for the stabilization of the cell’s state when it is to be passed on to the neighbors that are in the switch phase. The release phase and the relax phase support the cell’s gradual preparation for a new switch.

**Figure 2:**
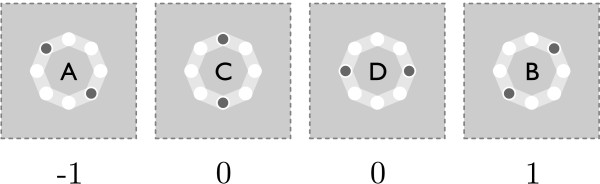
**Clocking scheme.** The clock cycle governing the pipeline transmission through a QCA. It is based on four phases, switch [0, $\frac{1}{4}$), hold [$\frac{1}{4},\;\frac{1}{2}$), release [$\frac{1}{2},\;\frac{3}{4}$), and relax [$\frac{3}{4}$, 1). Indices 0–3 indicate the clock zones, governed by a phase shifted original clock signal C_0_, so that when a cell in clock zone 0 is in the hold phase, a cell in clock zone 1 is in the switch phase, a cell in clock zone 2 is in the relax phase, and a cell in clock zone 3 is in the release phase.

Recent research [10] showed that the correct behavior of tQCA logic gates requires a synchronized data transfer, achievable through a pipelined architecture based on the adiabatic clock. The four-phased nature of the clock signal allows any tQCA to be decomposed to smaller stages or subsystems, controlled by phase shifted signals, each defining its own clock zone (Figure 2). With the correct assignment of cells to clock zones (clocking scheme), the direction of data flow can be controlled. The latency of a QCA circuit is determined by the number of clock zones along its critical path. A sequence of four clock zones causes the delay of one clock cycle. Consequently, minimizing the number of clock zones leads to better designs [29].

## Methods

Simulations were conducted following the same methods as outlined in the work of Pečar et al. [10]. Inter-layer interaction was analyzed as a two-cell system of one well-polarized cell and one observed cell placed directly one over the other but on separated layers. Through a series of sequential steps, a transition from the initial state to neutral and then from the neutral state to the final state is applied to the well-polarized cell, and the response of the observed cell is computed. The response is computed by numerical diagonalization of a tight-binding Hubbard-type Hamiltonian, where quantum dots are represented as sites, and the degrees of freedom internal to the quantum dots are ignored. The same set of parameters was used as in the work of Pečar et al. [10], with the inter-layer distance equal to that between the neighboring cells.

The behavior of the wire crossing was assessed using the intercellular Hartree approximation, as in the work of Pečar et al. [10]. A single simulation consists of the circuit’s total delay time discrete time steps. We use 200 time steps per clock cycle. At each time step, the ground state of the QCA is found by iteratively solving for the ground state of each cell. The ground state of a cell (observed cell) is calculated under the influence of the states of all other cells in the QCA which are momentarily treated as well-polarized. In turn, each of the QCA cells is chosen as the observed cell, so their states change. This process is iterated until the QCA relaxes, and no further change in any of the cells is observed (i.e., until the QCA reaches its ground state at the corresponding time step). At every simulation, an initial state is applied to cells marked as input cells (X1,X2) and the simulation run for the corresponding total delay time of the QCA circuit. This is when the cells marked as output cells (Y1,Y2) are in the hold phase, and their states are treated as valid. We simulated all possible combinations of initial states.

## Results and discussion

### Multi-layer interaction

QCA processing is based on inter-cell interaction, where the state of a cell influences the states of its neighbors and vice versa. The same applies for inter-layer interaction. The cell that is closest to the observed cell has the largest influence on the observed cell’s state. In a multi-layer case, two cells are closest when placed directly one over the other, i.e., on the same location but on separate layers.

**Figure 3:**
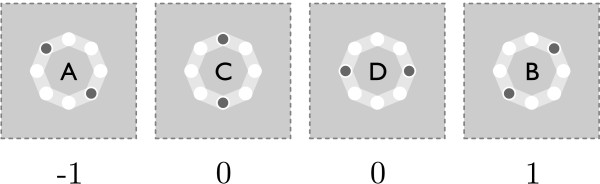
**Inter-layer interaction.** State transitions of the well-polarized cell and the corresponding response functions of the observed cell. Six state transitions are presented, namely A→B, A→C, A→D, B→C, B→D, and C→D. For all cases throughout a series of sequential steps, a transition from the initial state to neutral and then from the neutral state to the final state is applied to the well-polarized cell, and the response of the observed cell is computed. For each transition, there are four graphs depicting the density correlation function _*P*S_for states A, B, C, and D. The lighter curve (blue) is for the well-polarized cell, and the darker curve (orange) is the response of the observed cell. Notice that states A, B, C, and D of the well-polarized cell induce states B, A, D, and C in the observed cell, respectively.

Figure 3 presents six state transitions of the well-polarized cell and the corresponding response functions for the observed cell. The results were obtained by numerical diagonalization of a tight-binding Hubbard-type Hamiltonian, where quantum dots are represented as sites, and the degrees of freedom internal to the quantum dots are ignored. The same set of parameters was used as in the work of Pečar et al. [10], with the inter-layer distance equal to that between the neighboring cells. Reverse transitions are not presented as they are symmetrical to those presented. Observing the graphs, it can be noticed that the observed cell saturates very quickly, and the response function is highly nonlinear for the initial and final states and almost flat for the other two. However, the observed cell does not assume the same state as it would if the two cells were on the same layer. State A in the well-polarized cell induces state B in the observed cell, B induces A, C induces D, and D induces C (for the last two cases interestingly as it would if the two cells were on the same layer). When the states are interpreted as logic values this translates to logic negation since C and D both represent logic value 0, A logic value −1, and B logic value 1.

A negation of the transferred logic value occurs by moving from one layer to the other. When designing a wire crossing, this has no real effect as eventually the transferred logic value will be negated once more upon moving back to the original layer. In the case when processing is to be performed on different layers, however, this fact has to be kept in mind. For states C and D, it presents no real problem, as they both represent the same logic value, and alternating between the two states is achieved through simple addition of another adjacent cell. For states A and B, which represent two opposite logic values (−1 and 1, respectively), this, however, means adding an inverter (which in its simplest form could be just one cell displaced diagonally) or designing the processing element based on an inverted input value.

### Synchronized two-layer wire crossing

**Figure 4:**
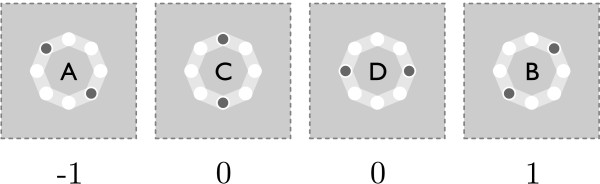
**Two-layer synchronized wire crossing.** The left image (L1:X1-Y1) shows the clocking scheme of wire X1-Y1. It is based on a diagonal pipelined transmission with blocks of two cells, producing a total delay of one clock cycle. The middle and right images show the clocking scheme of wire X2-Y2. The middle image (L1:X2-Y2) the part on layer L1; note that the clocking scheme is such that the active cells cause no interference with the cells carrying information of wire X1-Y1. The right image (L2:X2-Y2) shows the part on layer L2, where the same concept of diagonal pipelined transmission is used as for line X1-Y1.

Figure 4 presents a two-layer synchronized wire crossing that achieves the most compact wire crossing possible. Typically, it will be employed when two wires running parallel to one another have to be swapped. There are only two layers in this design, with the inter-layer distance equal to that between neighboring cells. Our experiments showed that this inter-layer distance is also the most robust one. The behavior of the wire crossing was assessed using the intercellular Hartree approximation, as in the work of Pečar et al. [10].

The total delay of the crossing is one clock cycle. The four phases are used so as to keep the distance between active cells (neighboring cells that are currently in the hold or switch phase) as large as possible, as well as to achieve robust inter-cell transfers. Active cells on the two layers are never directly one over the other, although this would not present a real issue as long as enough cells are active in the same instant. Reducing the number of active cells makes them more susceptible to inter-layer crosstalk, all due to the highly nonlinear inter-cell interaction.

The line, marked X1-Y1, travels in a diagonal fashion upwards on layer L1. This is achieved in one clock cycle (four phases), with blocks of two cells, so that the same state that is input to the first cell, marked X1, appears on the last cell, marked Y1, after a delay of one clock cycle.

The line, marked X2-Y2, travels first vertically from layer L1 to layer L2, then in a diagonal fashion downwards, and back vertically from layer L2 to layer L1, again all in one clock cycle. This ensures that the state that is input to the first cell, marked X2, appears on the last cell, marked Y2, after a delay of one clock cycle.

**Figure 5:**
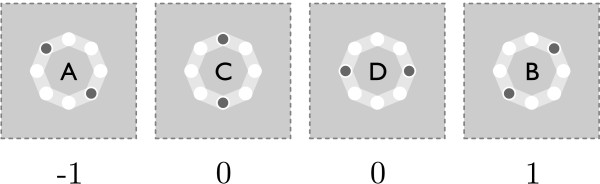
**Two-layer synchronized three wire crossing.** Application of the two-layer synchronized wire crossing concept to a three wire crossing. The total delay is 1.75 clock cycles.

Figure 5 presents the two-layer synchronized wire crossing concept applied to a three wire crossing. The total delay is 1.75 clock cycles, but the outputs of all three wires are synchronized, meaning the total latency is the same for all three wires.

## Conclusions

Due to the specifics of the ternary quantum-dot cell, the basic building block of ternary quantum dot cellular automata, coplanar wire crossings are not possible. In this article, we present a two-layer synchronized wire crossing; a wire crossing that uses such clocking schemes that the effective latency is equal for both wires (one clock cycle). In addition, the clocking schemes allow for a two-layer design. They override the requirement for additional layers, whose sole purpose is to prevent possible crosstalk.

Our current research is devoted to the study of synchronized two-layer wire crossings that consume fewer clock cycles as well as tile-based solutions, what we find to be one of the more promising approaches for QCA design in general.

## Competing interests

The authors declare they have no competing interests.

## Author’s contributions

ILB designed the two-layer synchronized wire crossing and the corresponding clocking scheme. He also prepared this manuscript. PP helped in the design of the clocking scheme, performed the simulations, and provided assistance through literature research for the preparation of the manuscript. Both authors read and approved the final manuscript.
